# Self-Assembly and Electronic States of an Electron Acceptor Molecule on a Metal Substrate

**DOI:** 10.1021/acsnano.6c06816

**Published:** 2026-08-11

**Authors:** Biswajit Pabi, Roberto Robles, Nicolas Lorente, Richard Berndt, Alexander Weismann

**Affiliations:** † Institut für Experimentelle und Angewandte Physik, 9179 Christian-Albrechts-Universität zu Kiel, 24098 Kiel, Germany; ‡ Centro de Física de Materiales CFM/MPC (CSIC-UPV/EHU), 20018 Donostia-San Sebastián, Spain; § Donostia International Physics Center (DIPC), 20018 Donostia-San Sebastián, Spain

**Keywords:** TCNQ, bilayer, organometallic network, SOMO-SUMO, scanning tunneling microscopy

## Abstract

Self-assembled monolayers and bilayers of the electron acceptor molecule tetracyanoquinodimethane (TCNQ) were investigated on Pb(100) using low-temperature scanning tunneling microscopy (STM) at 4.5 K and density functional theory (DFT) calculations. The monolayer exhibits an unusually low molecular density. In contrast, both the first and second layers in the bilayer adopt a packing density twice that of the monolayer. Our modeling reveals that Pb atoms, apparently released from step edges, stabilize the bilayer structure. The structural transition is accompanied by a pronounced change in the electronic structure: the bilayer hosts a singly occupied molecular orbital (SOMO) and its corresponding unoccupied partner (SUMO), arising from a spin-splitting of the lowest unoccupied molecular orbital (LUMO) observed in the monolayer.

## Graphical abstract

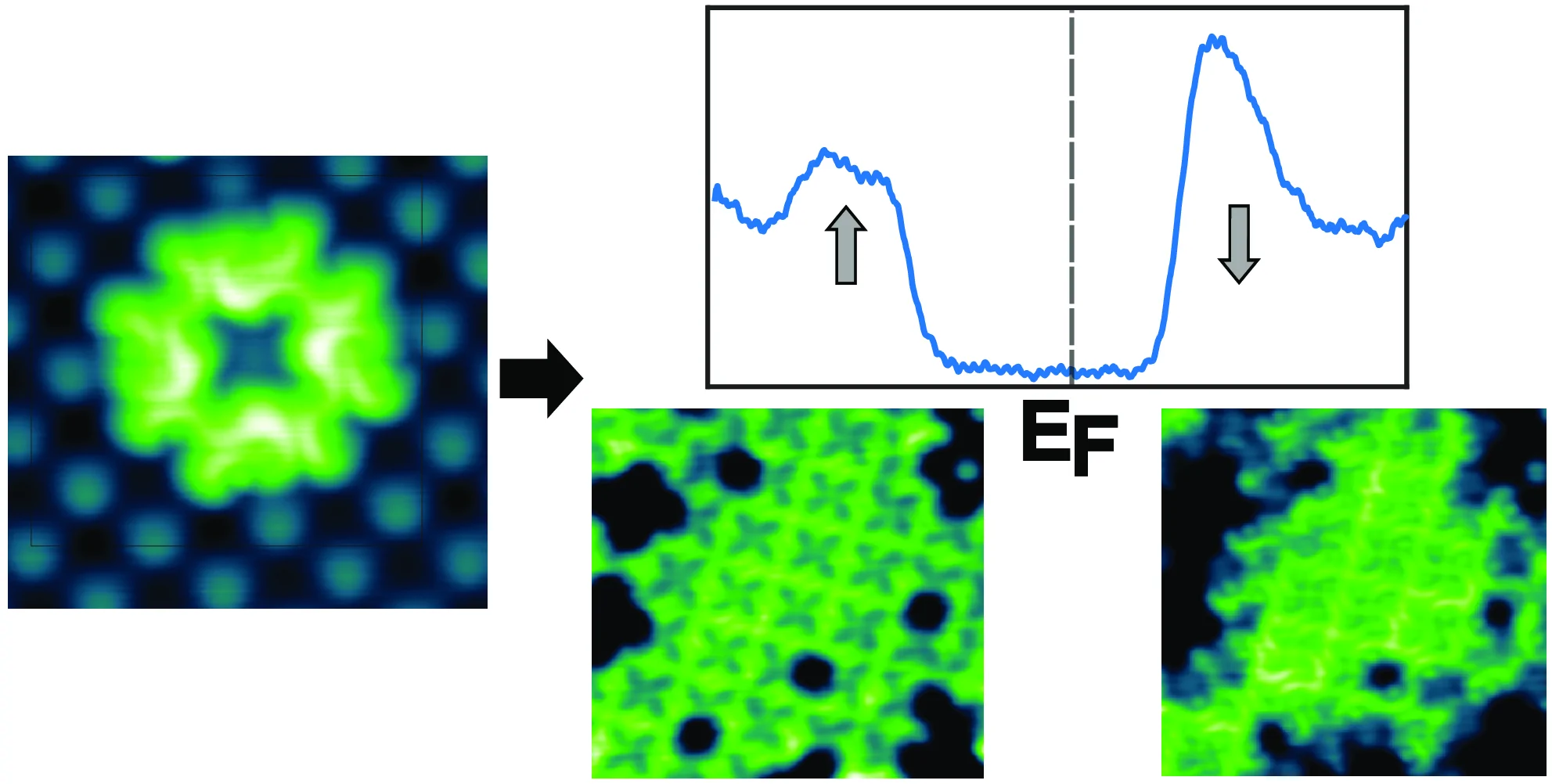

## Introduction

Organic heterostructures composed of electron-accepting and -donating molecules exhibit rich electronic and optical properties that make them attractive for a wide range of technological applications.^1–4^ Charge-transfer molecules adsorbed on surfaces are particularly important for realizing molecular conductors,^5^ tuning the work functions of organic electronic devices,^6,7^ and enabling chemical modifications of graphene.^8,9^

Meanwhile, the interface between organic molecules and ferromagnetic substrates, the so-called spinterface, has received much attention for spin-dependent properties that may be relevant for device applications.^10–13^

Tetracyanoquinodimethane (TCNQ) is a strong electron acceptor that plays a central role in forming organic metals when paired with donor molecules.^14^ Its high acceptor strength originates from its highly electrophilic cyano end groups. Previous studies have shown that TCNQ interacts strongly with Ag^15–17^ and Cu^18–22^ surfaces. In the case of Cu, this strong interaction modifies both the charge state and the geometry of the adsorbed TCNQ molecules and even induces surface reconstructions that affect the molecular arrangement. In contrast, the interaction with Au surfaces is comparatively weak and involves minimal charge transfer.^23–26^ Weak charge-transfer-induced magnetism has also been observed for TCNQ adsorbed on graphene/Ru(0001).^27^

TCNQ coverages on metals beyond the first monolayer remain largely unexplored by scanning tunneling microscopy (STM): early work by Schott and Ward on TTF-TCNQ, a donor–acceptor pair, under ambient conditions is a notable exception.^28^ In general, studies of molecular multilayer films with single-molecule resolution are still scarce.^29^ For TCNQ, however, thicker films are of particular interest due to the strong electrophilicity of the molecule. While the first layer is directly coupled to the electron reservoir of the substrate, additional layers no longer have direct access to this charge supply, raising fundamental questions about their structure and electronic properties.

Here, we combine STM and density functional theory (DFT) to investigate the self-assembled layers of TCNQ on Pb(100). While the molecular density in the monolayer is unusually low, the bilayer exhibits twice the density in both the first and second layers. Pb substrate atoms, which apparently emerge from the step edges, stabilize the bilayer structure. The structural transition is accompanied by a pronounced change in the electronic structure: the lowest unoccupied molecular orbital (LUMO) observed in the monolayer splits in the bilayer into a singly occupied molecular orbital (SOMO) and its corresponding unoccupied partner (SUMO). A spin-dependent splitting of the LUMO of second-layer molecules has been reported in discussing the magnetoresistance of a molecular film on a ferromagnetic substrate.^30^ Surprisingly, we find that a bilayer of TCNQ on nonferromagnetic lead exhibits a split density of states of the top layer while the layer directly on the substrate does not.

## Results and Discussion

### TCNQ Self-Assembly on Pb(100)

Submonolayer amounts of TCNQ molecules deposited onto Pb(100) at room temperature spontaneously organize into layers extending several hundred nanometers across the surface (Figure 1a). No isolated molecules are observed, suggesting significant intermolecular interactions and a sufficiently low diffusion barrier for the Pb(100) substrate.

**Figure 1:**
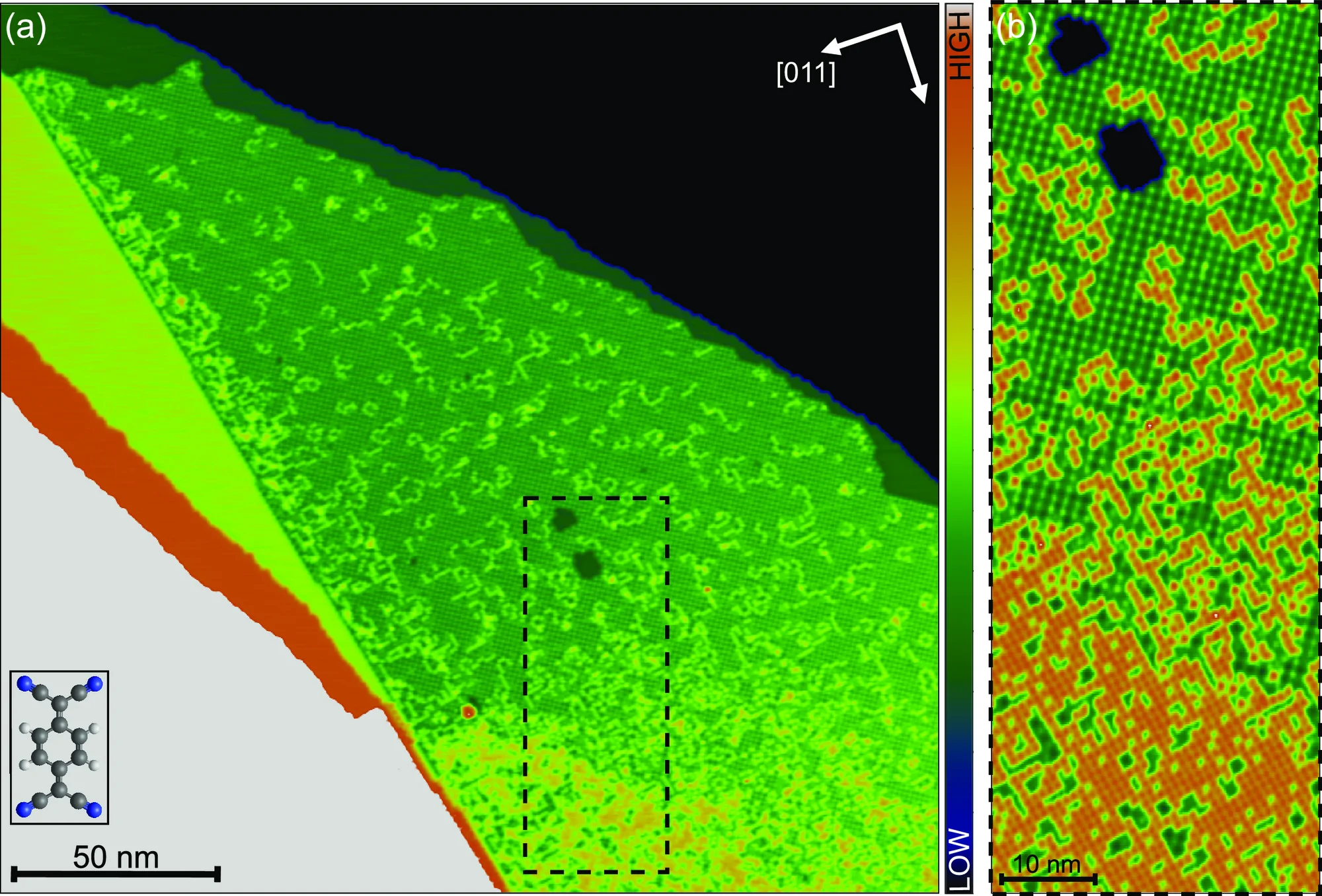
Constant-current topographs. (a) Overview image (230 × 230 nm^2^, *V* = 0.5 V, *I* = 50 pA) of a submonolayer coverage of TCNQ on Pb(100) showing multiple Pb terraces separated by monatomic steps. The ⟨011⟩ directions of the substrate are indicated by white arrows in the top-right corner. A schematic of the TCNQ molecule is shown in the lower-left corner, with gray, white, and blue spheres representing carbon (C), hydrogen (H), and nitrogen (N) atoms, respectively. (b) Higher-resolution image (95 × 35 nm^2^, *V* = −0.2 V, *I* = 100 pA) of the dashed rectangular area highlighted in (a). Green and orange features correspond to first- and second-layer molecules, respectively. The two black regions near the top represent the exposed substrate.

Closer inspection of an island (Figure 1b) reveals the presence of both a first (green) and a second (orange) molecular layer. The second layer is densely packed in the lower part of the image, while it is considerably sparser in the upper region. In the following, we refer to these two configurations as dense and sparse second layers. Notably, the density of second-layer molecules is highest near the ascending substrate step (left edge of the TCNQ island) and decreases toward the opposite edge of the molecular island. This pronounced density gradient suggests that Pb adatoms may play an active role in the self-assembly process, a scenario that is supported by the DFT calculations presented below. Deposition at cryogenic temperatures could, in principle, isolate the effect of Pb adatoms by suppressing their detachment from kink sites; however, it would also freeze molecular mobility. This would likely yield disordered aggregates rather than the highly ordered, adatom-stabilized thermodynamic phases formed at room temperature.

The ascending step is unusually straight and predominantly oriented along a ⟨032⟩ direction of the Pb crystal. This step geometry is atypical for clean Pb(100) surfaces and indicates that molecular adsorption induces a faceting of the atomic terraces. A similar reorganization of Pb(100) in response to molecular adsorption was previously reported.^31^

The unit cell of the first layer is square and commensurate with the underlying Pb(100) lattice. It is rotated by 58° with respect to the substrate unit cell, contains a single molecule, and has a lattice parameter of 1.25 nm [this superstructure may be described by the matrix $\left(\begin{array}{ll} 2 & 3\\ -3 & 2 \end{array}\right)$ ]. A tentative structural model derived from the experimental data is shown in Figure 2a. Notably, the molecular density of this layer is unusually low, leaving a substantial fraction of Pb atoms uncovered.

**Figure 2:**
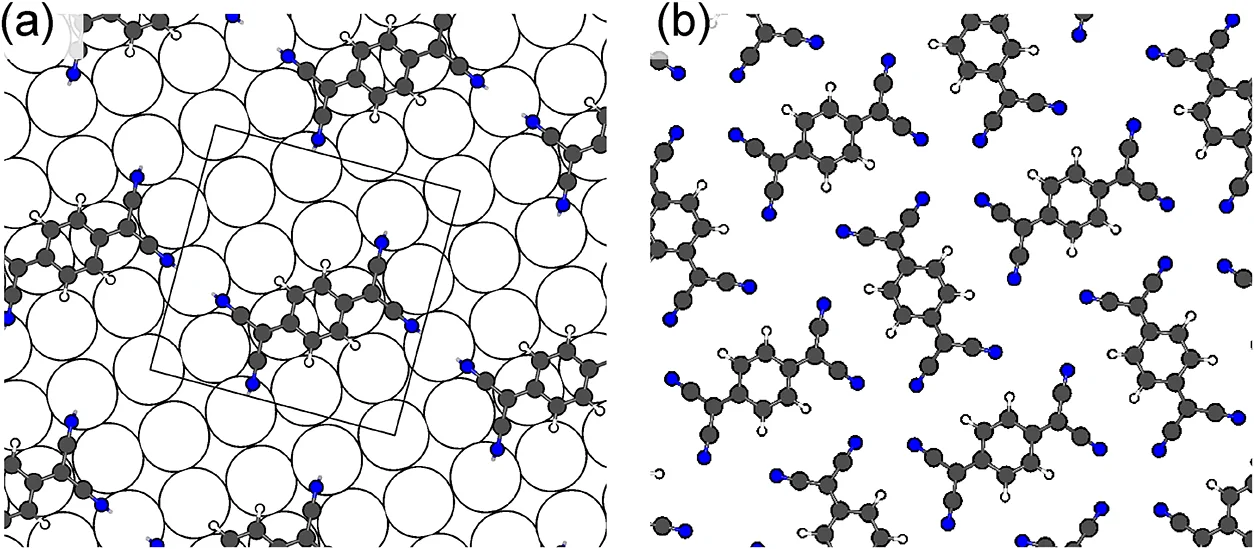
Tentative structure models. (a) Monolayer structure of TCNQ on Pb(100). The azimuthal orientations of the molecules are arbitrary, as the experimental STM images only show featureless protrusions. (b) Top layer of the bilayer structure. The azimuthal orientations are chosen to reproduce the rectangular appearance of the second-layer molecules observed in STM.

In the dense regions of the second layer, the density of molecular protrusions is doubled compared to the first layer. The corresponding unit cell has the same lateral dimensions as that of the first layer but is rotated by 45° relative to it and contains two molecules instead of one. A rectangular shape of the molecular protrusions is clearly resolved, revealing the azimuthal orientation of the molecules and supporting the structural model shown in Figure 2b. As expected, mirror-symmetric islands were also observed in other surface regions.

While the adsorption sites of molecules in the first layer cannot be unambiguously determined from the available experimental data, the positions of molecules in the sparse second layer can be readily identified. These molecules preferentially align along the diagonal of the first-layer unit cell and occupy the bridge sites of the underlying molecular lattice. In the case of the dense second layer, attempts to probe the underlying first layer by STM manipulation were unsuccessful, likely because of the stability of the molecular network.

### Layer-Resolved Spectroscopy

Figure 3 shows differential conductance (d*I*/d*V*) spectra acquired on a molecule in the first layer (red), a molecule in the sparse second layer (green), and a molecule in the dense second layer (blue). The spectra of the first layer and sparse second-layer molecules each exhibit a pronounced peak at −1.24 and −0.68 V, respectively. In contrast, spectra recorded on the dense second layer reveal two distinct peaks on either side of the Fermi level, at −0.91 and 0.62 V.

**Figure 3:**
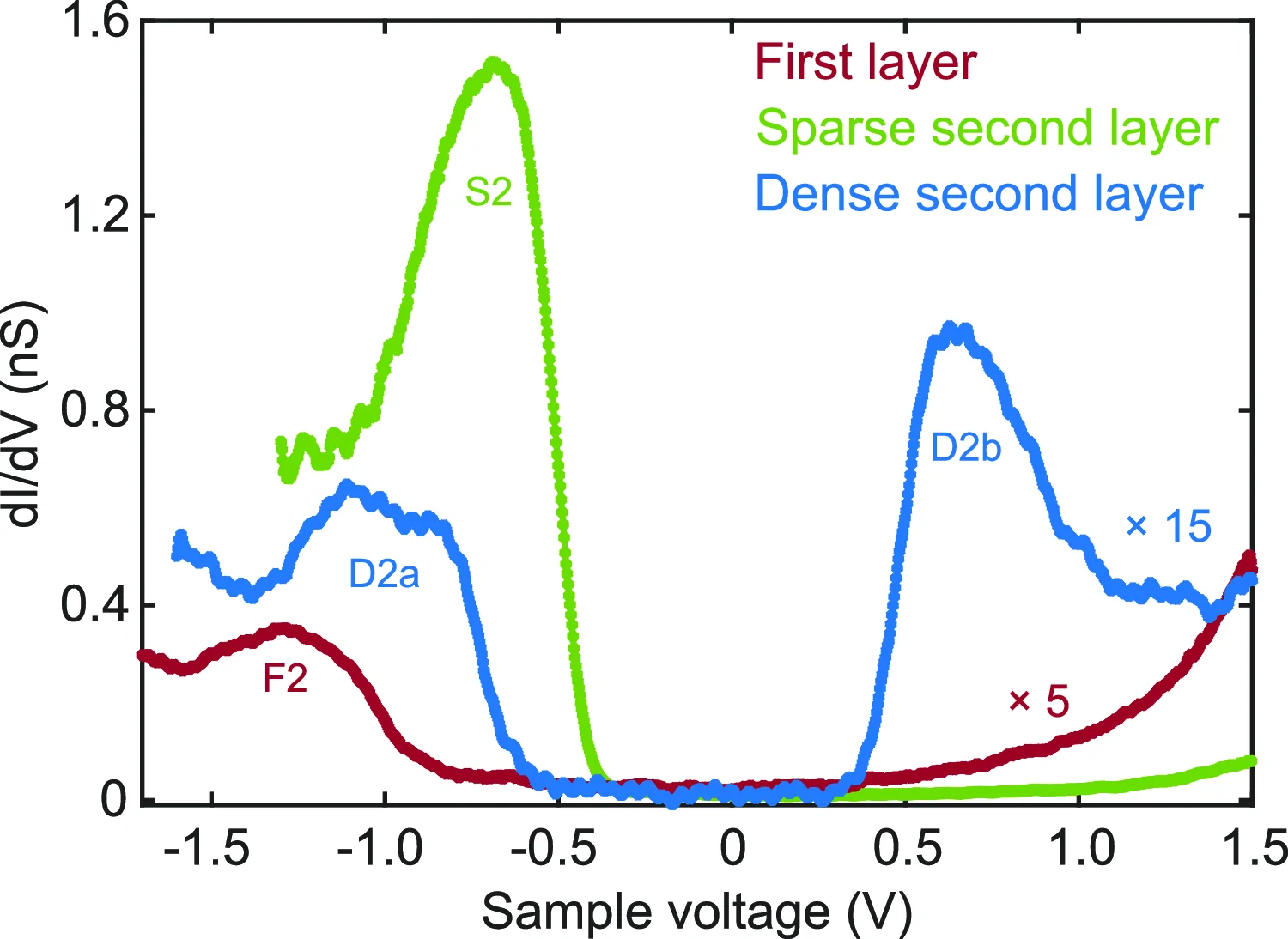
Constant-height d*I*/d*V* spectra. Red, green, and blue curves correspond to measurements above molecules in the first layer, the sparse second layer, and the dense second layer, respectively. The tip position was fixed prior to spectroscopy using a set point of *V* = 1.5 or 1.6 V and *I* = 100 pA. For clarity, the blue and red spectra have been multiplied by factors of 15 and 5, respectively.

To access a broader voltage range, we additionally recorded conductance spectra in constant-current mode (Figure 4). Beyond the range shown, the tunneling junction became unstable. Besides the feature around 0 V inherent to this measurement mode, the spectra display two, three, and four maxima for the first-layer (red), sparse second-layer (green), and dense second-layer (blue) molecules, respectively. The corresponding peak energies are summarized in Table 1, together with those obtained from constant-height measurements. Reference spectra obtained on the substrate are shown in the Supporting Information, Figure S1.

**Figure 4:**
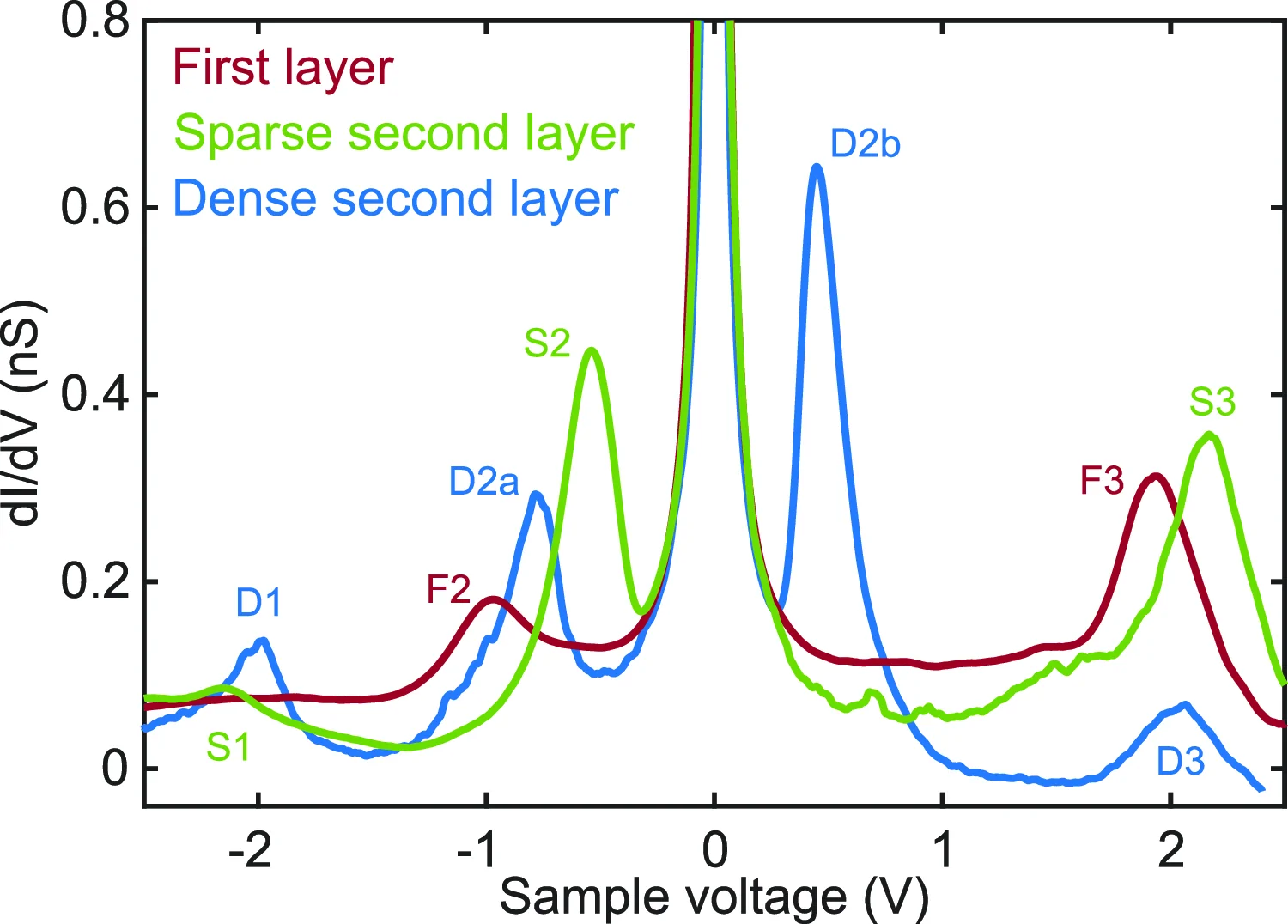
Constant-current d*I*/d*V* spectra. The same color scheme as in Figure 3 is used. The measurements started at *V* = ±10 mV using *I* = 100 pA.

**Table 1:** Peak Positions (in V) Extracted from d*I*/d*V* Spectra Measured in Constant-Height and Constant-Current Modes on Different Molecular Layers^a^

layer	constant height	constant current
first	–1.24 (F2)	–1.00 (F2), +1.94 (F3)
sparse second	–0.68 (S2)	–2.16 (S1), −0.55 (S2), +2.17 (S3)
dense second	–0.91 (D2a)	–1.99 (D1), −0.79 (D2a)
	+0.62 (D2b)	+0.44 (D2b), +2.04 (D3)

a Labels in parentheses indicate the peak assignments shown in Figures 3 and 4.

For a meaningful comparison between data from both modes of measurement, it is important to recall that spectral maxima in constant-current measurements are slightly shifted toward zero bias and appear sharper than those obtained at constant height.^32^ Consequently, the constant-current maxima at −1.00, −0.55, −0.79, and 0.44 V correspond to the constant-height peaks observed at −1.24, −0.68, −0.91, and 0.62 V, respectively. Comparing the first and the sparse second layer, all spectral features are shifted to higher energies in the sparse second layer. The peak at −2.16 V appearing in the constant-current spectrum of the sparse second layer likely lies outside the accessible range for the first-layer measurements. In contrast, comparison of the dense and sparse second layers suggests that the peaks at −0.79 and 0.44 V in the dense configuration originate from a splitting of the −0.55 V feature observed in the sparse second layer. The remaining peaks at −1.99 and 2.04 V closely match the corresponding features at similar energies in the sparse second-layer spectra. Taken together, the d*I*/d*V* spectra reveal an evolution of the electronic structure with molecular packing and layer configuration. In particular, the densely packed second layer exhibits a pronounced splitting of spectral features near the Fermi level. In the following, we investigate this effect by analyzing the bias dependence of the STM images.

### Bias-Dependent Imaging

Figure 5 shows constant-current STM images of TCNQ assemblies recorded at sample voltages in the range ±1.5 V, covering one electronic state of the first and sparse second layers and two states of the dense second layer. The appearance of the first-layer molecules remains largely unchanged over the entire voltage range, maintaining a characteristic rectangular shape. In contrast, the images of second-layer molecules exhibit a pronounced bias dependence. When the sample voltage *V* is close to the spectral peaks of sparse (S2) and dense (D2a, D2b) second layer, the molecular contrast (Figure 5b,d,f) evolves from a simple rectangular shape into a more complex pattern (this pattern is chiral and the opposite handedness was observed in other islands) consisting of two protrusions and two stripe-like features (hereafter referred to as the *two-dots–two-stripes* pattern, red symbols in Figure 5b,d,f). The observed patterns closely resemble the spatial distribution of the gas-phase LUMO of TCNQ (Supporting Figure S4), as previously reported for TCNQ adsorbed on Au(111) and graphene/Ru(0001)^23,27^ and are inconsistent with those of other frontier orbitals. Note that the *two-dots–two-stripes* features appear higher in the STM image than the rectangular ones (Supporting Table S1) as expected from the involvement of electronic resonances. Intriguingly, the images of occupied and unoccupied states share the identical nodal plane of the gas-phase LUMO, suggesting a common underlying origin.

**Figure 5:**
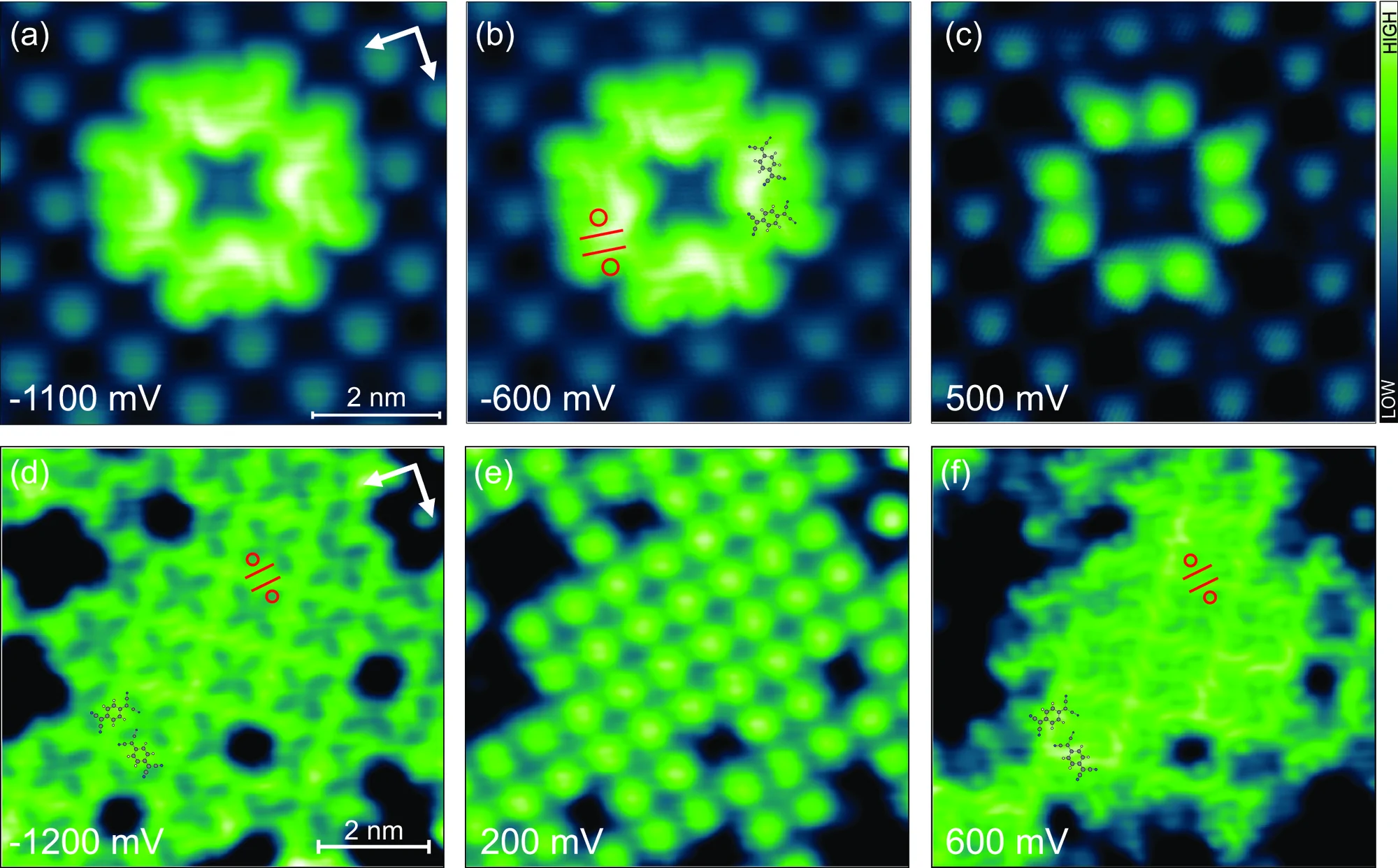
Bias dependence of constant-current images. (a–c) Images (7 × 7 nm^2^, *I* = 100 pA) of an island containing both first-layer and sparse second-layer molecules, recorded at the indicated sample voltages. The substrate ⟨011⟩ directions are indicated by white arrows in (a). It should be noted that edges of the molecular unit cell enclose an angle of 32° with the close-packed substrate directions, i.e., the molecular array is mirror symmetric to the structure of Figure 1. While second-layer molecules appear as featureless protrusions at positive bias, they exhibit distinct intramolecular contrast (*two-dots–two-stripes*, red patterns in (b, d, f)) at negative voltages. The contrast of first-layer molecules remains largely unchanged across the applied voltages. (d–f) Images (8 × 8 nm^2^, *I* = 200 pA) of the dense second layer at different sample voltages *V* with an overlaid molecular model. Patterns resembling the *two-dots–two-stripes* motif (red pattern) are observed at −1.2 and 0.6 V, whereas no intramolecular contrast is visible at 0.2 V, corresponding to the energy gap between the states resolved in the d*I*/d*V* spectra. Note that the molecular unit cells in panels (d–f) have the same orientation as in Figure 1, while the unit cells in (a–c) are their mirror image.

Even without the calculations presented later, the experimental data already suggest that the original S2 state of the sparse bilayer configuration splits into singly occupied (D2a) and singly unoccupied (D2b) molecular orbitals (SOMO and SUMO) located on either side of the Fermi level in the densest packed arrangement. Spatially resolved spectroscopy further indicates that the LUMO-derived orbital of the first layer lies significantly below the Fermi level, suggesting substantial charge transfer from the substrate to the first layer. Reduced charge transfer to the sparse second layer shifts this state closer to the Fermi level. In the dense second layer, an additional peak appears above the Fermi level that has no corresponding feature in either the monolayer or sparse bilayer configuration. Moreover, the spatially resolved conductance patterns of the states below and above the Fermi level are highly similar, suggesting that both originate from the same gas-phase LUMO and represent a spin-split, partially occupied orbital.

### Modeling Results

Density functional theory (DFT) calculations were performed to elucidate the adsorption geometry and electronic structure of TCNQ on Pb(100). We first consider the single-layer, in which the molecules adopt a relatively low surface density. Considering all the molecules to have the same orientation, the most stable configuration is shown in Figure 6a. In this configuration, the molecules adsorb with the quinoid ring centered at bridge sites of the Pb surface lattice, with the molecular long axis aligned parallel to a substrate ⟨011⟩ (close-packed) direction. However, in the experimental images of the monolayer, the molecules look like featureless protrusions, and their azimuthal orientation can not be determined. Therefore, a configuration where all the molecules do not have the same orientation is also consistent with the experimental observations. Taking this into consideration, we find that a structure where the molecules have alternating orientations is energetically preferred by 9 meV per molecule (Figure 6b). Here, the molecules are adsorbed at hollow sites and along the [001] and [010] directions. Other possibilities are shown in the Supporting Information (Figure S5). In these configurations, Pb atoms of the surface lattice coordinated to nitrogen atoms are slightly displaced out of the surface plane. Such substrate relaxation may account for the relatively low density, similar to the TNCQ-induced surface reconstruction of Cu(100).^18^

**Figure 6:**
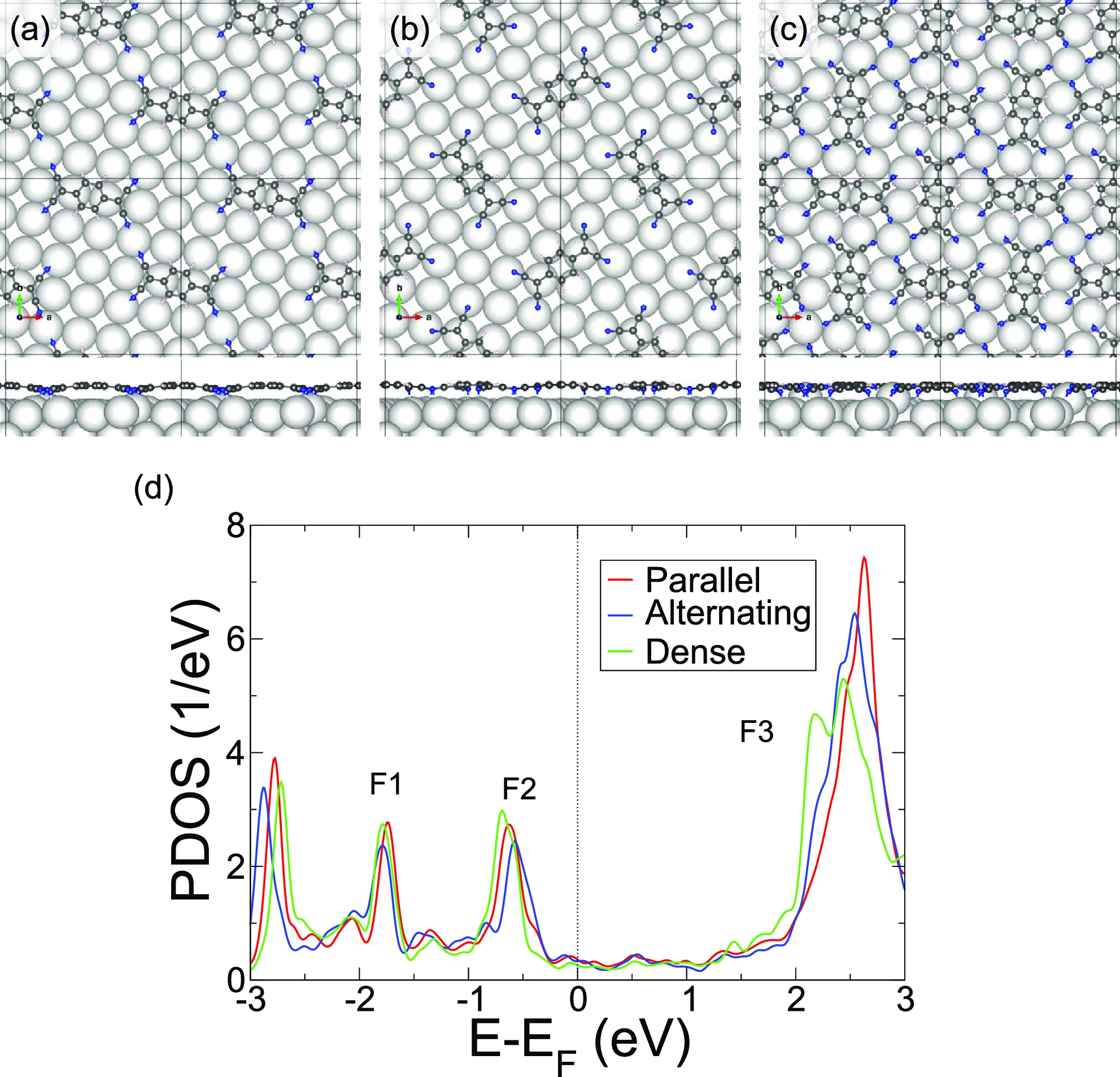
Models and electronic structure of the first molecular layer. Top and side views of the most stable configurations of a sparse first layer of TCNQ molecules adsorbed on Pb(100) in (a) parallel and (b) alternating orientations. (c) Top and side views of a dense first layer of TCNQ adsorbed on bridge sites in an alternating arrangement. Large white spheres represent Pb atoms. Black lines separate unit cells. (d) Projected density of states (PDOS) of a TCNQ molecule in (a–c).

The projected densities of states (PDOS) for the two molecular configurations are very similar (Figure 6d), exhibiting pronounced peaks at −2.8, −1.8, −0.5, and 2.5 eV. The latter two peaks (denoted as F2 and F3) are consistent with experimentally observed states at −1 and 2 eV, assuming that DFT overestimates the orbital energies by 0.5 eV.

A monolayer with a doubled molecular density was also modeled (Figure 6c). In this configuration, four cyano groups are coordinated to some substrate Pb atoms, which as a consequence are displaced outward (see side view of Figure 6c). The total energy of this structure is in fact 98 meV lower compared with the model of Figure 6b. However, STM images consistent with this structure were only rarely observed and were confined to small surface areas. We hint that the interaction of the molecules with the steps, which is not considered in the DFT calculations, may favor the structure of Figure 6b, possibly through kinetic effects.

The PDOS shown in Figure 6d (compare with the red curves in Figures 3 and 4) closely resembles that of the sparse monolayer. As in the configuration of Figure 6a, the TCNQ molecules are centered at bridge sites of the substrate. The small lateral displacements required to shift adsorption from hollow to bridge sites cannot be resolved experimentally because of the broad molecular features in STM images.

Molecules in the first layer are hybridized with the substrate, and they get charged by approximately 1.4 e^–^/molecule from the Pb surface.

Several candidate molecular bilayer structures were examined theoretically. Placing a dense second layer atop a sparse first layer leads to pronounced molecular ruffling, resulting in simulated STM images that are inconsistent with experimental observations (Supporting Information, Figure S6). In contrast, a flatter second layer is found over the dense first layer. The most stable solution is shown in Figure 7a, with second-layer molecules located between first-layer molecules. Other configurations are shown in the Supporting Information, Figure S7. However, this solution still shows some buckling, and the simulated STM images do not completely agree with the experimental images. The second layer can be further stabilized by adding Pb adatoms to some hollow sites of the substrate. Including adatoms, the most stable configuration is shown in Figure 7b, while less favorable structures are presented in the Supporting Information, Figure S8. Now, second-layer molecules are located directly atop first-layer molecules, and some cyano groups of the second-layer molecules coordinate to the Pb adatoms.

**Figure 7:**
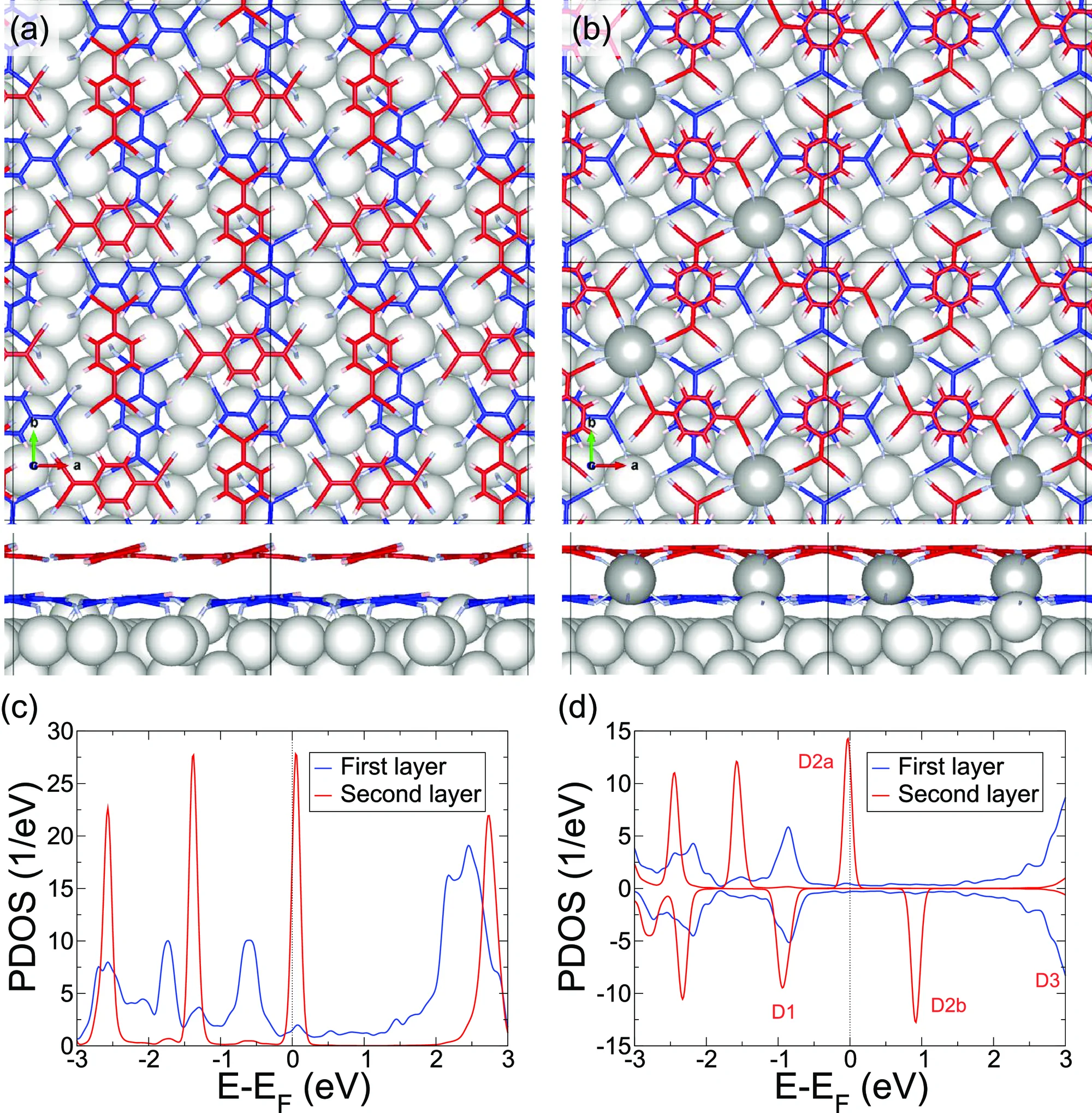
Model and electronic structure of dense second layer. (a, b) Top and side views of the dense bilayer, with schemes of first- and second-layer molecules shown in blue and red, respectively. In (a), the second-layer molecules are positioned between the first-layer molecules, while they are placed directly atop the first layer in (b), with additional adatoms (in gray) occupying hollow sites of the substrate. (c, d) Projected density of states (PDOS) of the TCNQ molecules corresponding to the structures shown in (a, b), respectively.

The charge transfer from the Pb substrate to the molecules in the first layer is around 1.3–1.4 e^–^/molecule with or without adatoms. However, the charge transfer to the molecules in the second layer is small without adatoms (0.1–0.2 e^–^/molecule) and much larger when Pb adatoms are added (0.6 e^–^/molecule). This is reflected in the PDOS. Without Pb adatoms (Figure 7c), the molecular orbitals of the second layer are shifted by 0.5–0.7 eV compared to the first layer, with the LUMO pinned at the Fermi energy due to the small charge transfer. When Pb adatoms are included (Figure 7d), the larger charge transfer induces a spin polarization, with the magnitude of the spin splitting depending on the choice of the DFT exchange and correlation functional (Supporting Information, Figure S9). Note that the D2a and D2b peaks in Figure 7d correspond to the peaks with the same labels in the blue curves of Figures 3 and 4, after accounting for the ≈0.5 eV overestimation of peak energies by DFT discussed above. Simulated STM images (Figure 8) of the structure with Pb adatoms (Figure 7d) calculated using the HSE06 functional show good agreement with experiment across a range of bias voltages, reproducing the characteristic *two-dots–two-stripes* patterns at both positive and negative bias, as well as the featureless protrusions observed in between. The *two-dots–two-stripes* patterns at negative (positive) voltages arise from singly occupied (unoccupied) molecular orbitals. Because adatoms are required to stabilize this structure, dense second-layer growth preferentially occurs near ascending step edges, where adatoms are readily available. In contrast, for the sparse second layer, the conditions necessary to produce a spin-polarized density of states are evidently not met, resulting in the *two-dots–two-stripes* pattern being observed only at negative bias.

**Figure 8:**
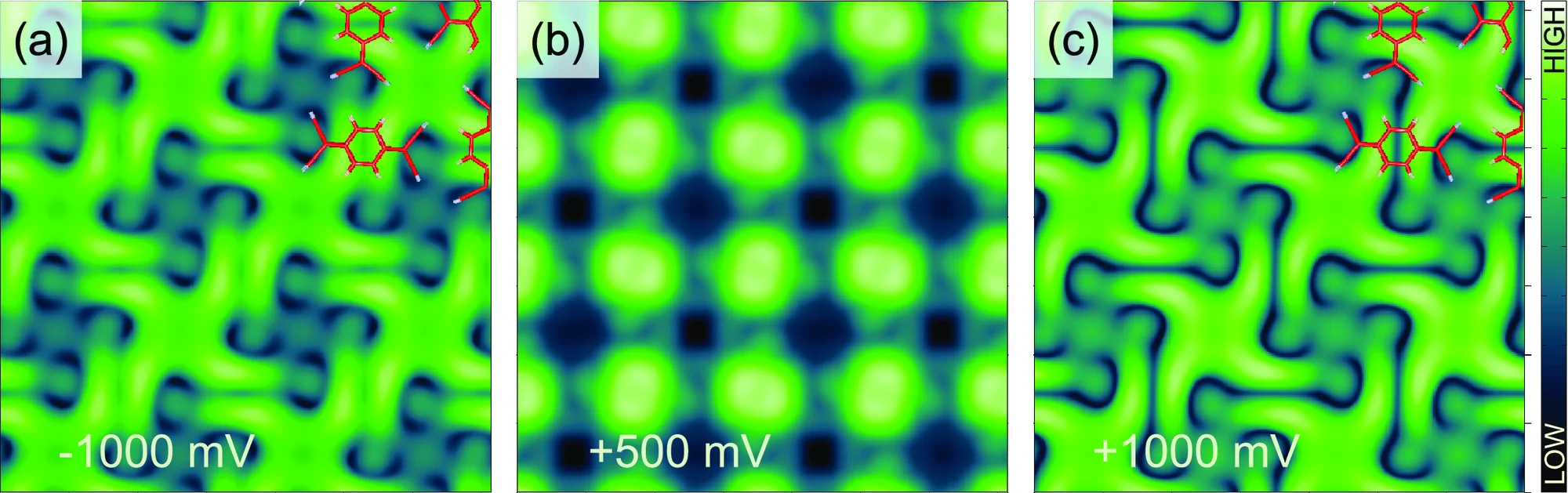
Simulated STM images of the dense second layer. The constant−current images were calculated for sample voltages of (a) –1000, (b) 500, and 1000 mV. The image area spans 3.6 × 3.6 nm^2^ with the molecular arrangement being identical to the one in Figure 7b. The HSE06 functional was used.

Our results demonstrate the realization of singly occupied orbitals in purely molecular layers directly supported on a metal substrate. The reduced packing density of the first layer is associated with a surface relaxation following molecular adsorption, as previously observed for TCNQ on Cu(100).^18^ Singly occupied orbitals have previously been observed from molecules adsorbed on insulating layers or on graphene.^33–38^ Although the appearance of singly occupied molecular orbitals in the dense second layer suggests a magnetic ground state, no Yu-Shiba-Rusinov states are detected in any of the assemblies (Supporting Figure S3).

This absence may be caused by the weak hybridization between the second-layer molecules and the substrate, implying a weak exchange interaction. As a consequence, the Kondo temperature is very low. In addition, possible YSR states may virtually coincide with the superconducting coherence peaks, thereby suppressing the appearance of a detectable magnetic spectroscopic fingerprint despite the presence of localized spin polarization.^39^ This interpretation is supported by the calculated spin density (Supporting Information, Figure S10), which appears largely confined to the second molecular layer and effectively isolated from the Pb surface.

## Conclusions

Overall, our study demonstrates that the interaction between TCNQ molecules and a Pb surface gives rise to distinct structural, electronic, and magnetic phenomena. In particular, these findings highlight the crucial role of interfacial charge transfer between electron-accepting molecules and metallic substrates in reshaping the molecular electronic structure, thereby enabling the design of hybrid architectures capable of exploiting the molecular magnetism.

## Methods

### Experiment

The Pb(100) substrate was prepared by repeated cycles of argon ion (Ar^+^) sputtering and subsequent annealing at temperatures up to 500 K. STM tips were cut from a Pb wire and sputtered in ultrahigh vacuum. TCNQ molecules were deposited from a Knudsen cell onto the Pb(100) substrate held at room temperature. The experiments were performed in ultrahigh vacuum using a custom-built STM at 4.5 K. Differential conductance (d*I*/d*V*) spectra were recorded by adding a sinusoidal modulation (frequency 833 Hz) to the bias voltage. For constant-current and constant-height spectroscopy, a modulation amplitude of 10 mV was used. High-resolution spectra near the Fermi level were acquired using a modulation voltage of 60 μV.

### Theory

Density functional theory calculations were performed using the VASP code.^40^ The projector augmented-wave method^41^ was applied together with a plane wave basis set with an energy cutoff of 500 eV. The PBE exchange and correlation functional^42^ was used in combination with the D3 method^43,44^ to treat missing van der Waals interactions. Selected calculations were done using the HSE06 hybrid functional^45^ using the PBE geometry. The Pb(100) surface was simulated using slabs with 5 layers of Pb and a vacuum layer of at least 20 Å. The Brillouin zone was sampled using a (3 × 3 × 1) *k*-grid. The coordinates of all atoms except the bottom two Pb layers were relaxed until all forces were smaller than 0.01 eV/Å. Charge transfers were determined by performing Bader analyses.^46^ STM images were simulated within the Tersoff-Hamann approximation^47^ using the method of Bocquet et al.^48^ as implemented in the STMpw code.^49^ Atomic and density plots were generated using the VESTA program.^50^

## Supplementary Material



## Notes

STM topographs of the dense first layer; spectroscopic data; and additional theoretical results, including modeled structures of the first layer and bilayer, projected density of states (PDOS), and spin density of the molecular bilayer (PDF)
