# Supplementary material for: Self-Assembly and Electronic States of an Electron Acceptor Molecule on a Metal Substrate
Source: ACS Nano. 2026 Aug 11;20(33):23330–7. doi: 10.1021/acsnano.6c06816 (PMC13523792; doi:10.1021/acsnano.6c06816)
Supplement: Supplementary file 1 [file nn6c06816_si_001.pdf]

Supporting Information for Self-Assembly and Electronic States of an Electron Acceptor Molecule on a Metal Substrate

Biswajit Pabi,† Roberto Robles,*,‡ Nicolas Lorente,‡,¶

Richard Berndt,*,† and Alexander Weismann*,†

†*Institut für Experimentelle und Angewandte Physik, Christian-Albrechts-Universität zu Kiel, 24098 Kiel, Germany*

‡*Centro de Física de Materiales CFM/MPC (CSIC-UPV/EHU), 20018 Donostia-San Sebastián, Spain*

¶*Donostia International Physics Center (DIPC), 20018 Donostia-San Sebastián, Spain*

E-mail: roberto.robles@csic.es; berndt@physik.uni-kiel.de; weismann@physik.uni-kiel.de

1. Experimental Results

1.1. Differential Conductance (dI/dV) Spectra of the Substrate

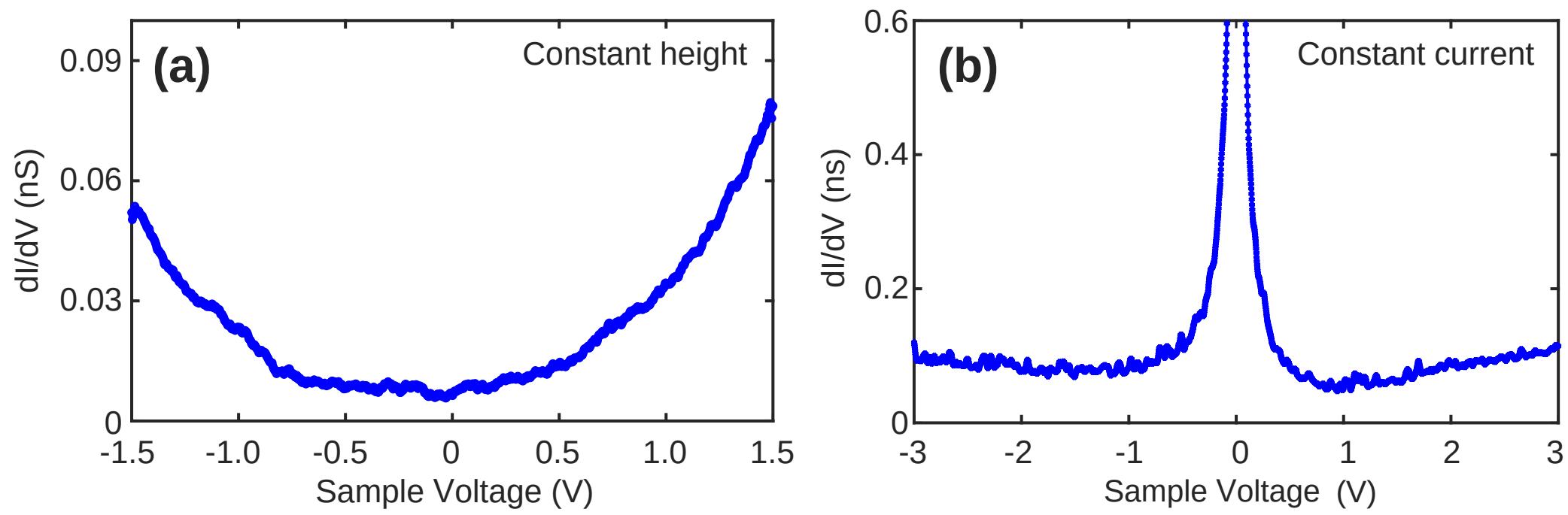

Figure S1: **dI/dV spectra of the substrate:** (a) constant height and (b) constant current spectra recorded above the Pb (100) substrate. The tip position was frozen prior to spectroscopy using a setpoint of $V = 1.5\,\text{V}$, $I = 100\,\text{pA}$ and $V = +10$ or $-10\,\text{mV}$, $I = 100\,\text{pA}$ for constant height and constant current spectra, respectively .

1.2. Locally Increased Packing Density of TCNQ in the First Layer

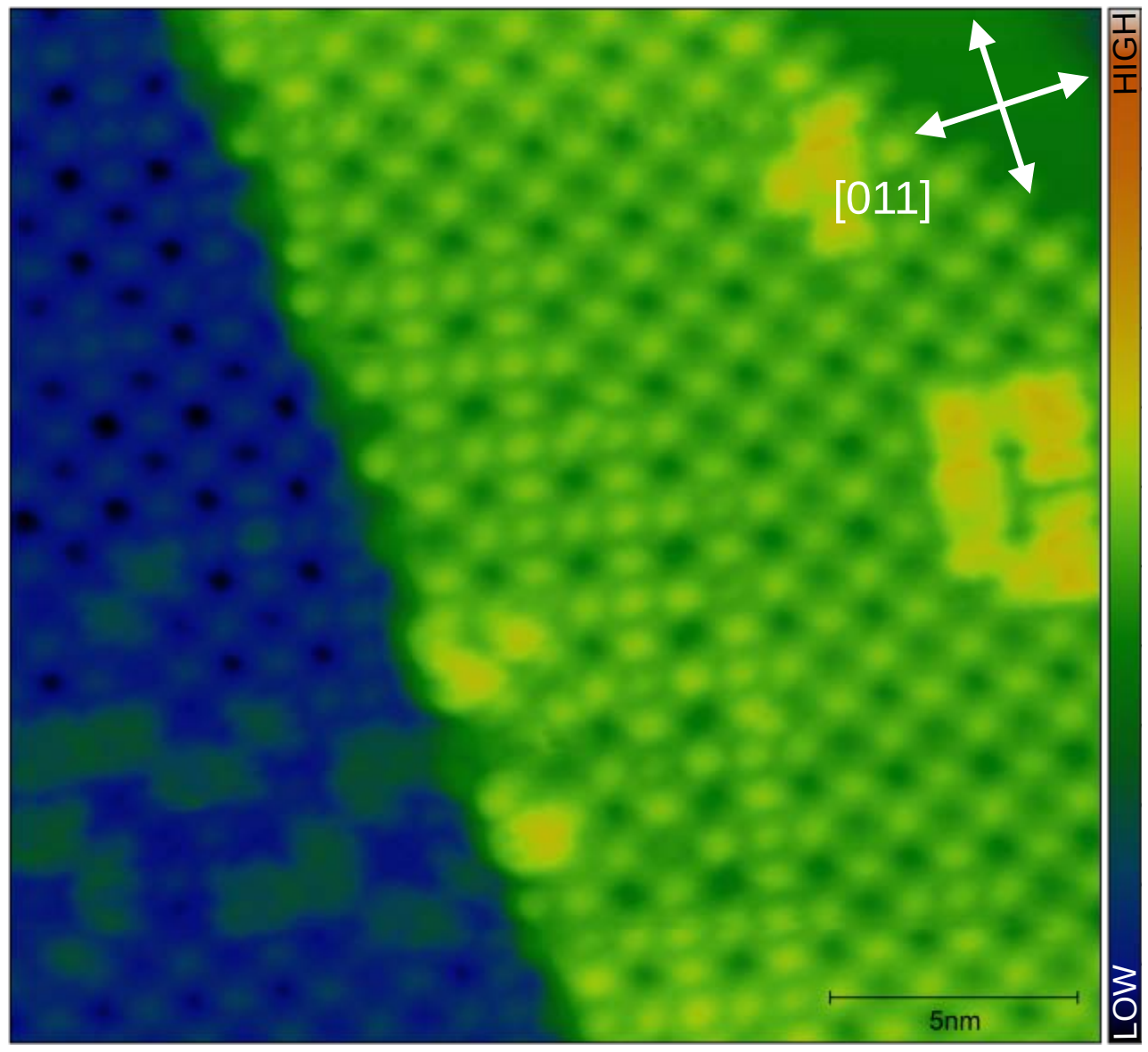

Figure S2: **Locally increased packing density within the monolayer:** Constant-current STM topograph ($22 \times 22\,\mathrm{nm}^2$, $V = -0.6\,\mathrm{V}$, $I = 100\,\mathrm{pA}$). The substrate [011] directions are indicated by white arrows in the top-right corner.

1.4. High Resolution dI/dV Spectra Near Fermi Level

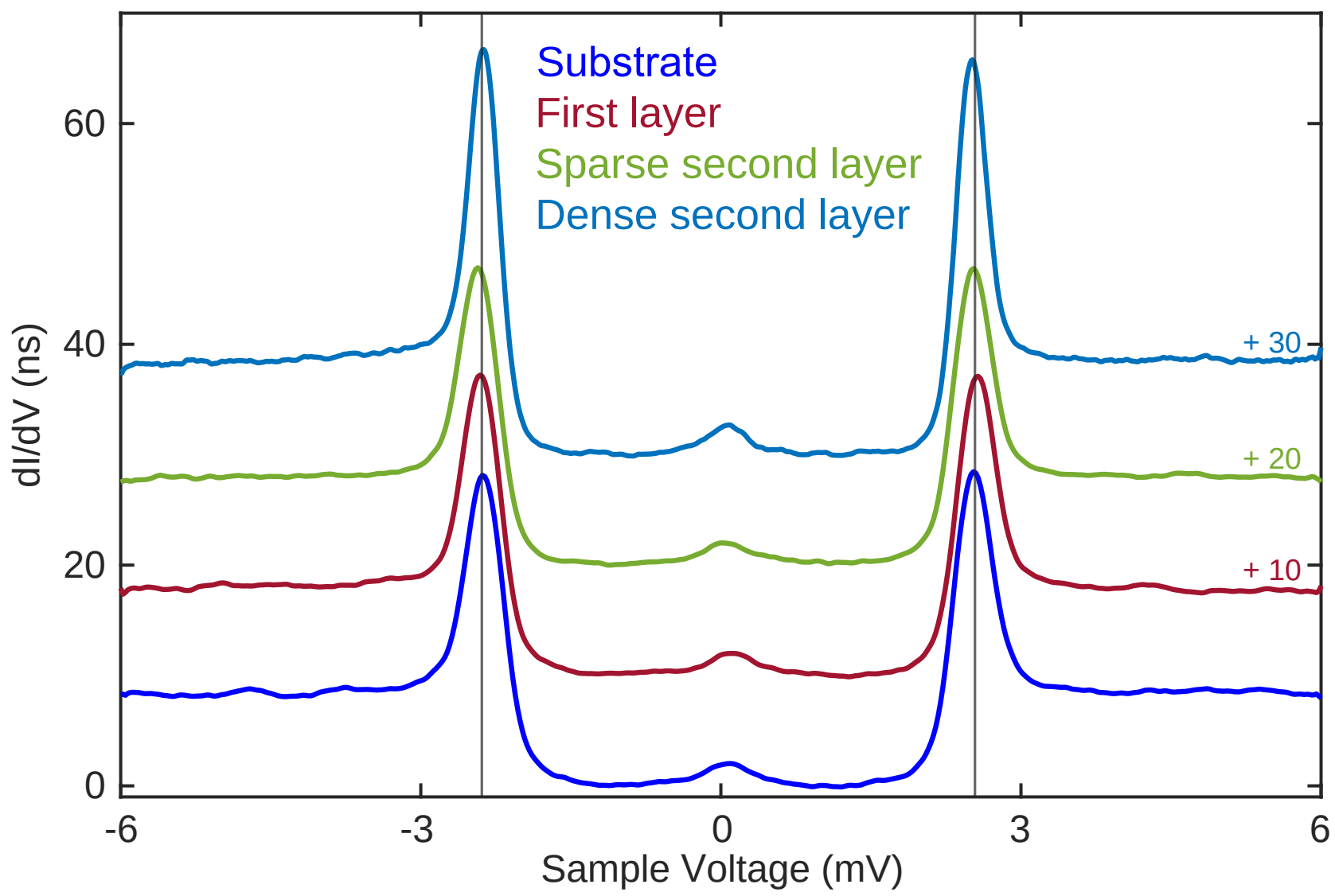

Figure S3: **High resolution dI/dV spectra close to the Fermi energy.** dI/dV spectra measured above the substrate (blue), a first layer molecule (dark red), a sparse second layer molecule (green) and dense second layer molecule (light blue). The tip position was frozen prior to spectroscopy using a setpoint of $V = 6\,\text{mV}$, $I = 100\,\text{pA}$. Gray lines indicate the edges of the superconducting gap. Dark red, green and light blue spectra are shifted vertically by 10, 20, and 30 nS for clarity.

1.5. Height Information of Molecular Layers

Table S1: Height of rectangular and *two-dots–two-stripes* features. Values in brackets indicate the corresponding voltages in V.

**Molecule with**	**Height in pm from surface to**		
	**First layer**	**Sparse second layer**	**Dense second layer**
Rectangular shape	200 (±1.2)	285 (+1.2)	285 (−0.4)
two-dots–two-stripes	**	392 (−1.2)	337 (+1.2)

2. Theoretical Results

2.1. Molecular orbitals from gas- phase DFT

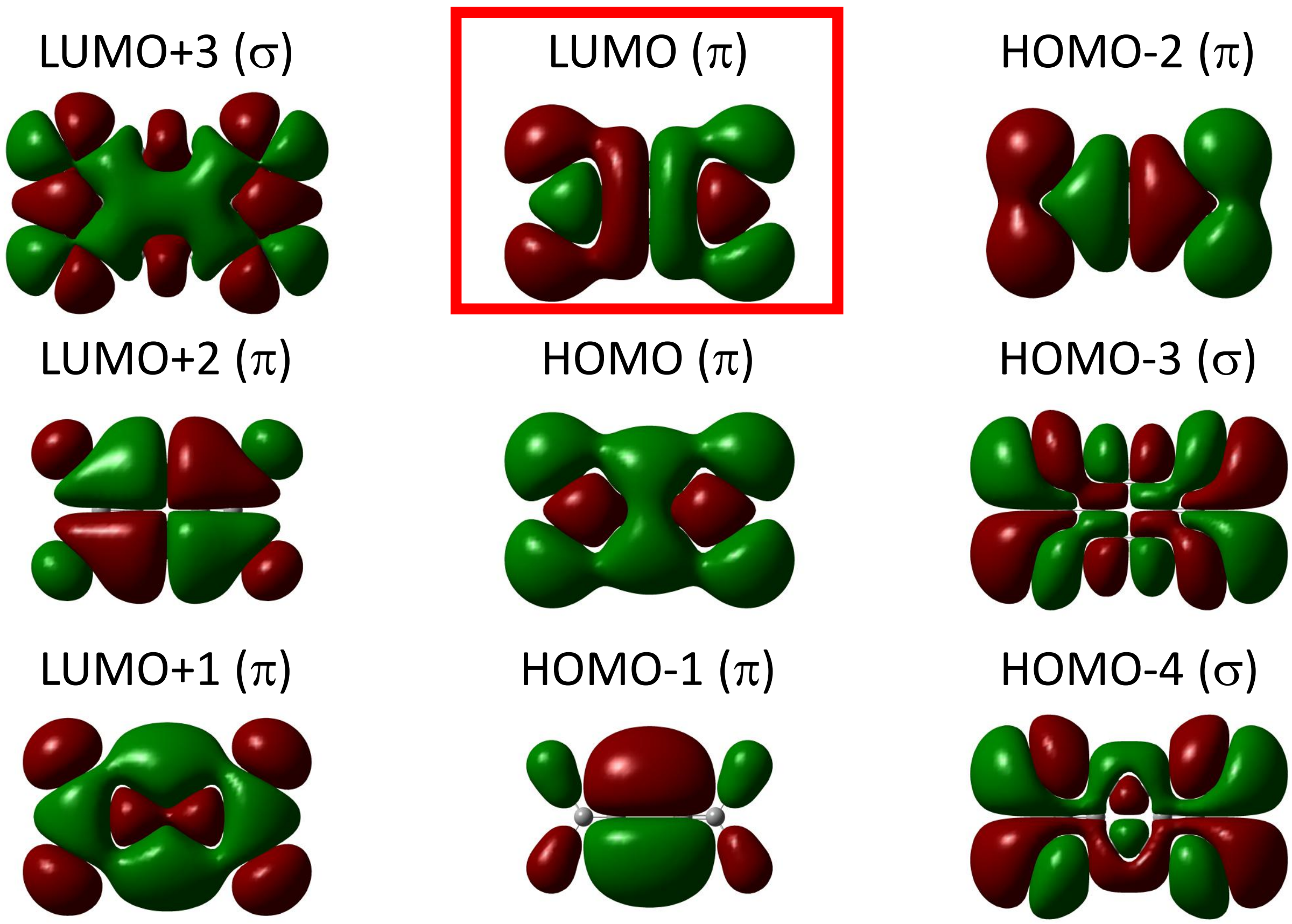

Figure S4: Wave-function isosurfaces of gas-phase TCNQ calculated using Gaussian.[1]

2.2. Configurations of the First Molecular Layer

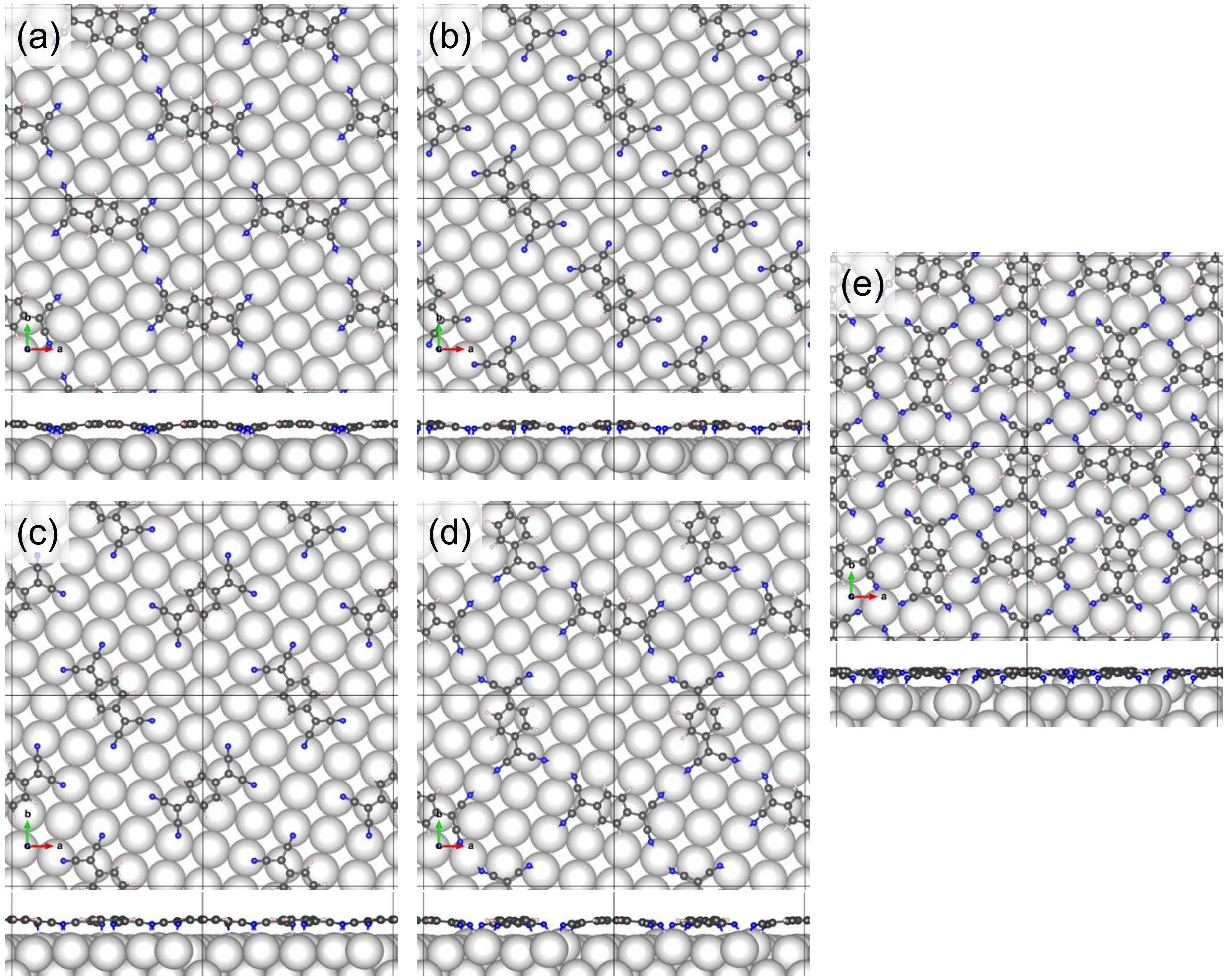

Figure S5: **Configurations of the first molecular layer** Panels a–d show top and side views of different configurations of a sparse first layer of TCNQ molecules adsorbed on Pb(100). (a) and (b) show parallel orientations and (c) and (d) show alternating orientations. The energy difference of these configurations in meV/molecule is (a) 9, (b) 26, (c) 0, and (d) 106. Panel (e) shows a top and a side view of a dense first layer.

2.3. Configurations of the Molecular Bilayer

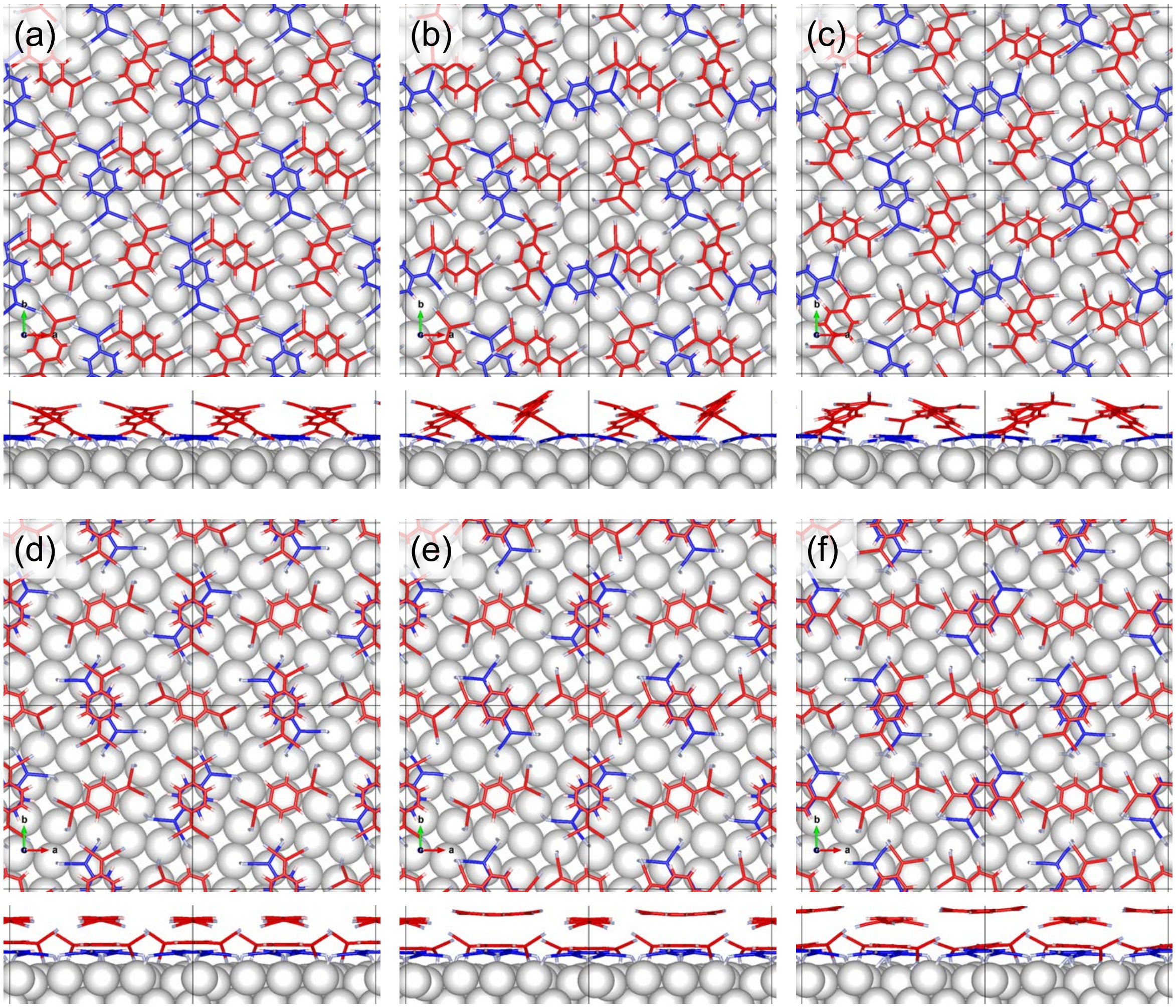

Figure S6: **Bilayers with a dense second layer placed on top of a sparse first layer.** Top and side views of different configurations of a dense second layer placed on top of a sparse first layer on Pb(100). TCNQ molecules initially in the first (second) layer are depicted as blue (red) schemes. Note that in the final configurations shown here some molecules originally in the second layer moved down and exhibit bonds between cyano groups and surface Pb atoms. As a consequence, the surface is ruffled. The energy differences of the configurations in meV/molecule are (a) 0, (b) 134, (c) 154, (d) 215, (e) 234, and (f) 249.

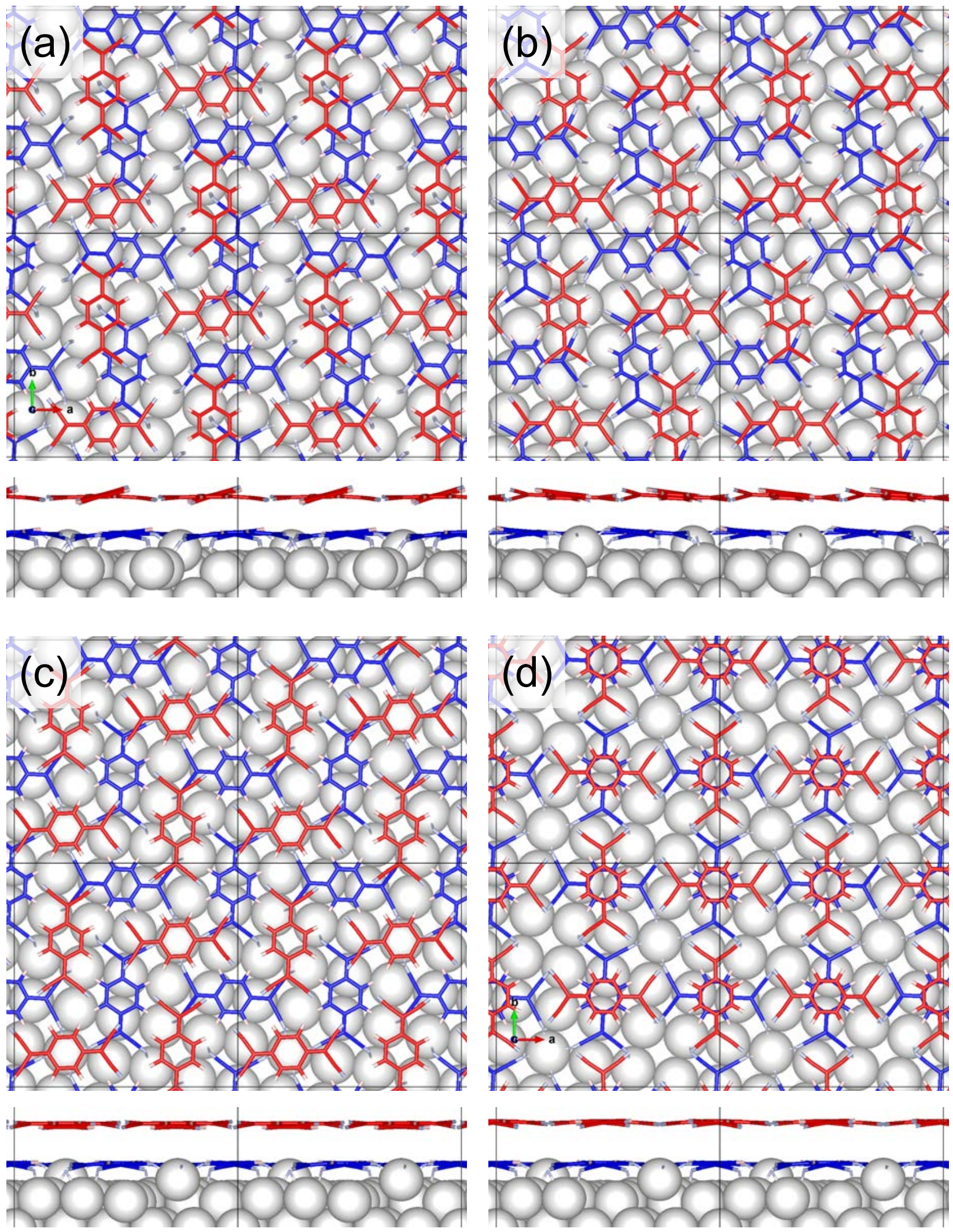

Figure S7: **Bilayers with a dense second layer placed on top of the dense first layer** Top and side views of different configurations of a dense second layer placed on top of a dense first layer on Pb(100). TCNQ molecules initially in the first (second) layer are depicted by blue (red) schemes. The energy differences of the configurations in meV/molecule are (a) 0, (b) 71, (c) 77, (d) 162.

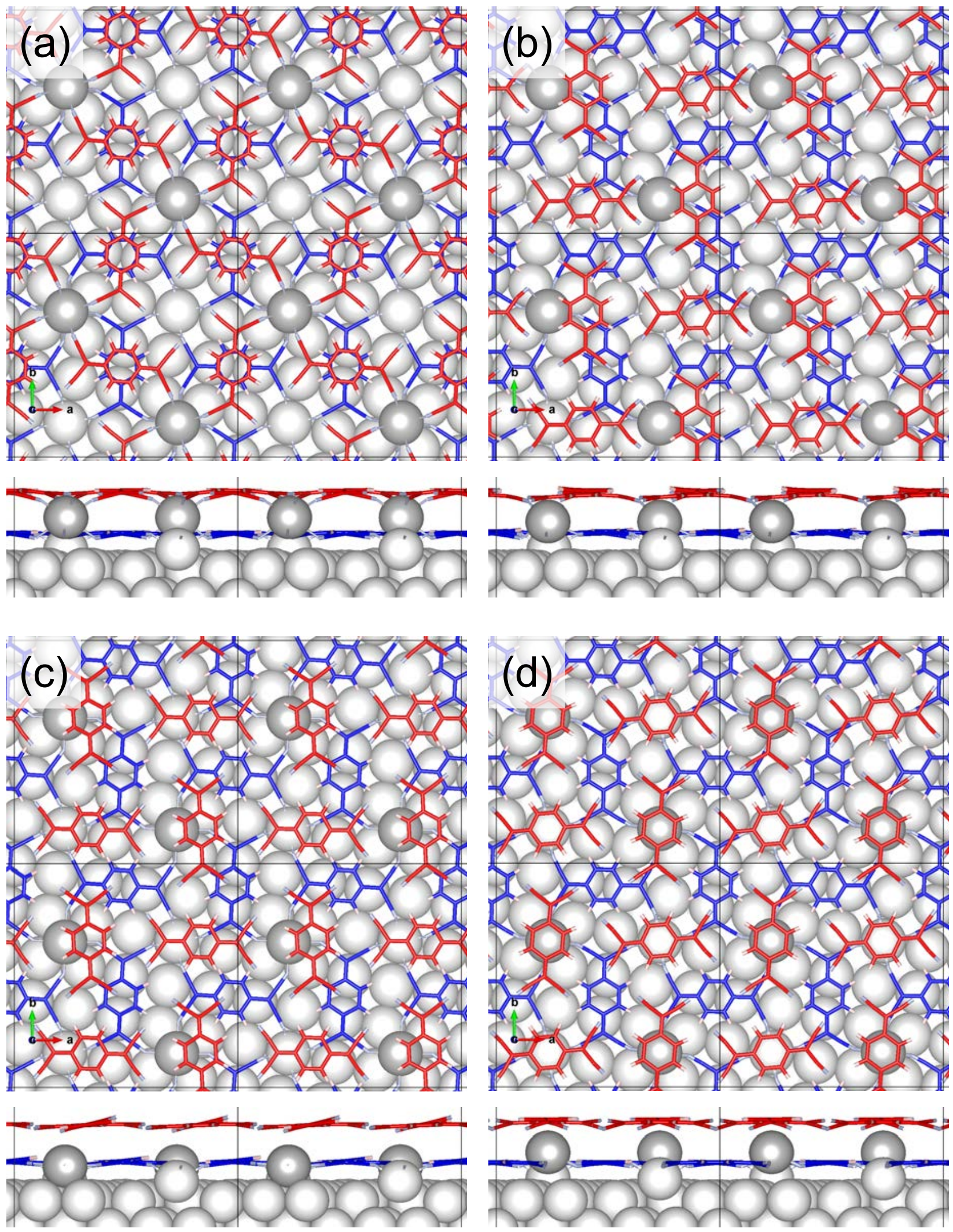

Figure S8: **Bilayers with a dense second layer placed on top of the dense first layer with Pb adatoms added** Top and side views of different configurations of a dense second layer placed on top of a dense first layer on Pb(100) with the addition of Pb adatoms. TCNQ molecules initially in the first (second) layer are depicted as blue (red) schemes. Pb adatoms are shown as grey spheres. The energy differences of the configurations in meV/molecule are (a) 0, (b) 297, (c) 380, (d) 400. In the most stable configuration (a), the Pb adatoms are coordinated to several cyano groups.

2.4. DFT calculations with Different Functionals

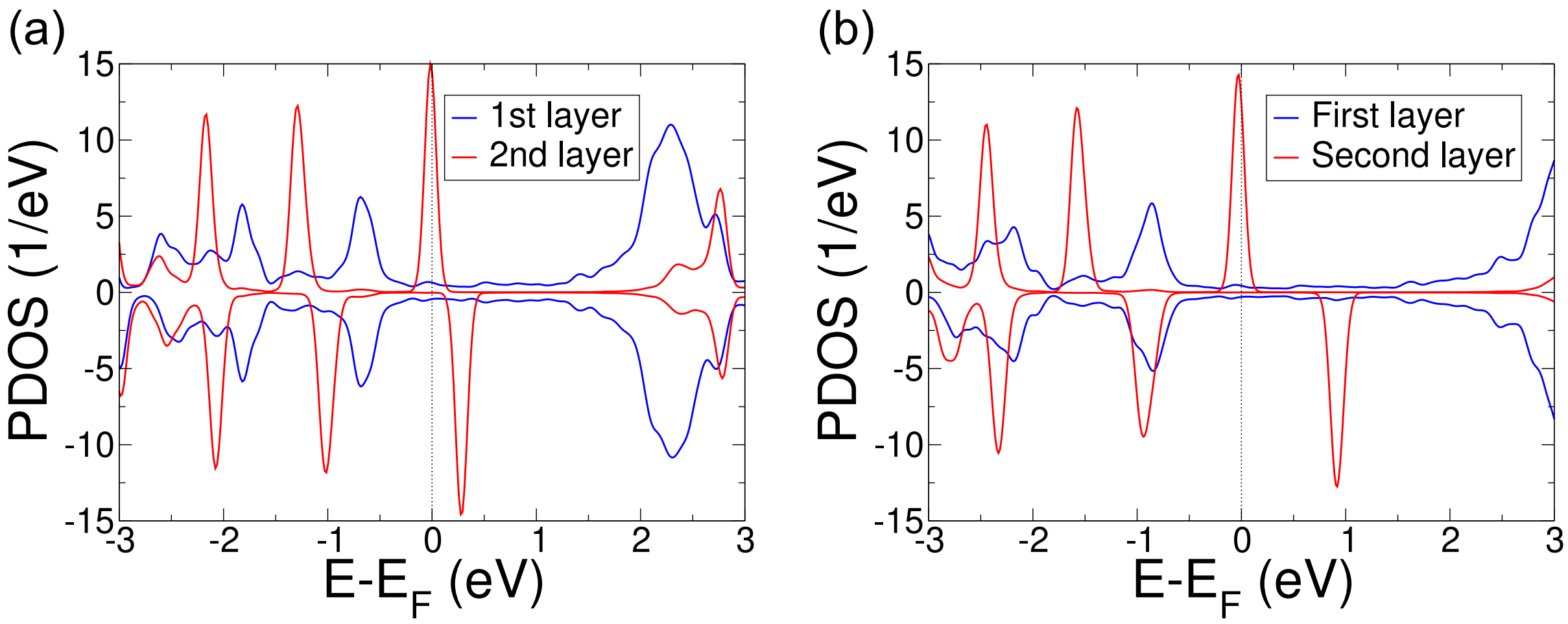

Figure S9: **PDOS of a molecular bilayer using two exchange-correlation functionals.** PDOS of the most stable configuration of the molecular bilayer including Pb adatoms using (a) the PBE functional and (b) the hybrid HSE06 functional. The spin-spitting is larger for the HSE06 functional. The molecules appear as featureless protrusions in the region of the gap between the SOMO and the SUMO. This region is only present for the hybrid HSE06 functional.

2.5. Charge Transfers and Adsorption Energies

Table S2: Charge transfers ΔN (in e^- per molecule), adsorption energies E_{ad} and $E_{\mathrm{ad,vdW}}$ (in eV per molecule) of TCNQ molecules in the configurations of Figures S5, S6, S7 and S8. $E_{\mathrm{ad,vdW}}$ indicates the vdW part of E_{ad}.

	ΔN (1st layer)				ΔN (2nd layer)					
	Mol 1	Mol 2	Mol 3	Mol 4	Mol 1	Mol 2	Mol 3	Mol 4	E_{ad}	$E_{\mathrm{ad,vdW}}$
Fig. S5a*	1.47	1.47	-	-	-	-	-	-	2.925	1.482
Fig. S5b	1.42	1.42	-	-	-	-	-	-	2.908	1.406
Fig. S5c*	1.43	1.43	-	-	-	-	-	-	2.934	1.332
Fig. S5d	1.46	1.46	-	-	-	-	-	-	2.828	1.498
Fig. S5e*	1.38	1.38	1.39	1.39	-	-	-	-	3.032	1.560
Fig. S6a	1.30	1.30	-	-	0.67	0.65	0.55	0.53	2.104	1.338
Fig. S6b	1.30	1.33	-	-	0.60	0.67	0.60	0.34	1.969	1.318
Fig. S6c	1.35	1.34	-	-	0.46	0.52	0.33	0.55	1.950	1.245
Fig. S6d	1.24	1.34	-	-	1.11	1.19	0.05	0.06	1.889	1.302
Fig. S6e	1.22	1.29	-	-	1.21	1.12	0.04	0.04	1.870	1.310
Fig. S6f	1.25	1.31	-	-	1.15	1.20	0.06	0.05	1.855	1.286
Fig. S7a*	1.26	1.26	1.32	1.32	0.12	0.12	0.15	0.15	2.086	1.370
Fig. S7b	1.26	1.26	1.24	1.24	0.12	0.12	0.13	0.13	2.016	1.317
Fig. S7c	1.31	1.31	1.31	1.31	0.11	0.11	0.12	0.11	2.010	1.329
Fig. S7d	1.34	1.33	1.33	1.33	0.11	0.11	0.11	0.11	1.924	1.299
Fig. S8a*	1.40	1.40	1.40	1.40	0.61	0.61	0.59	0.59	2.634	1.347
Fig. S8b	1.33	1.33	1.38	1.38	0.48	0.49	0.45	0.45	2.338	1.391
Fig. S8c	1.32	1.32	1.36	1.37	0.17	0.17	0.33	0.33	2.254	1.391
Fig. S8d	1.34	1.34	1.35	1.35	0.14	0.14	0.75	0.75	2.234	1.317

*Figures S5a, c, and e correspond to Figures 6a, b, and c. Figures S7a and S8a correspond to Figures 7a and b.

Table S2 shows the charge transfer and the adsorption energies of TCNQ molecules in the configurations of Figures S5, S6, S7 and S8. Since TCNQ is a strong acceptor, all the charge transfers are positive, meaning electron transfer from the Pb(100) surface to the molecules. Molecules in the first layer are directly over the Pb(100) surface. Their cyano groups are coordinated to Pb atoms of the surface and the hybridization is important. As a consequence the charge transfer is bigger than one electron per molecule (between 1.2 and 1.5 e^-/molecule). Molecules in the second layer show a larger variation of charge transfer. Some molecules in the configurations of Figure S7 d–f are quite isolated and show negligible charge transfers ($<$ 0.1 e^-/molecule). In the same cases, other molecules initially in the

second layer move down during the geometry optimization until they are in contact with the Pb substrate. Thus their charge transfers are equivalent to molecules in the first layer (1.1 to 1.2 e^-/molecule). For dense second layers on top of dense first layers (Figure S7) the charge transfers are small, around 0.1 to 0.2 e^-/molecule. In the presence of Pb adatoms the charge transfer of molecules in the second layer is, in general, much larger due to the hybridization with the Pb adatoms. For the most stable case (Figure S8a) every molecule in the second layer gets 0.6 e^-.

The adsorption energies E_{ad} shown in Table S2 are defined as

$$E_{\mathrm{ad}}[n \cdot (\mathrm{TCNQ})@\mathrm{Pb}(100)] = (n \cdot E[\mathrm{TCNQ}] + E[\mathrm{Pb}(100)] - E[n \cdot \mathrm{TCNQ}@\mathrm{Pb}(100)])/n,$$

where n is the number of adsorbed molecules, $E[\mathrm{TCNQ}]$ is the energy of one molecule, $E[\mathrm{Pb}(100)]$ is the energy of the Pb(100) surface, and $E[n \cdot \mathrm{TCNQ}@\mathrm{Pb}(100)]$ is the energy of the full system. $E_{\mathrm{ad,vdW}}$ indicates the vdW part of E_{ad}, where energies E in the expression above are substituted by the vdW energy within the D3 method. Adsorption energies for molecules in a sparse first molecular layer (Figure S5) are around to 3 eV/molecule, of which only 1.3 to 1.6 eV correspond to the vdW part. These values indicate significant chemical bonding. When second layer molecules are included (Figures S6, S7), the adsorption energy decreases to around 2 eV/molecule. Assuming that the adsorption energy for the molecules in the first layer of the bilayer is the same as for the first molecular layer ($\sim$3 eV/molecule), the adsorption energy for molecules of the second layer is around 1 eV per molecule. When Pb adatoms are included (Figure S8), the system is significantly stabilized and the adsorption energy increases to 2.6 eV/molecule for the most stable case.

2.6. Spin Density of the Bilayer Including Adatoms

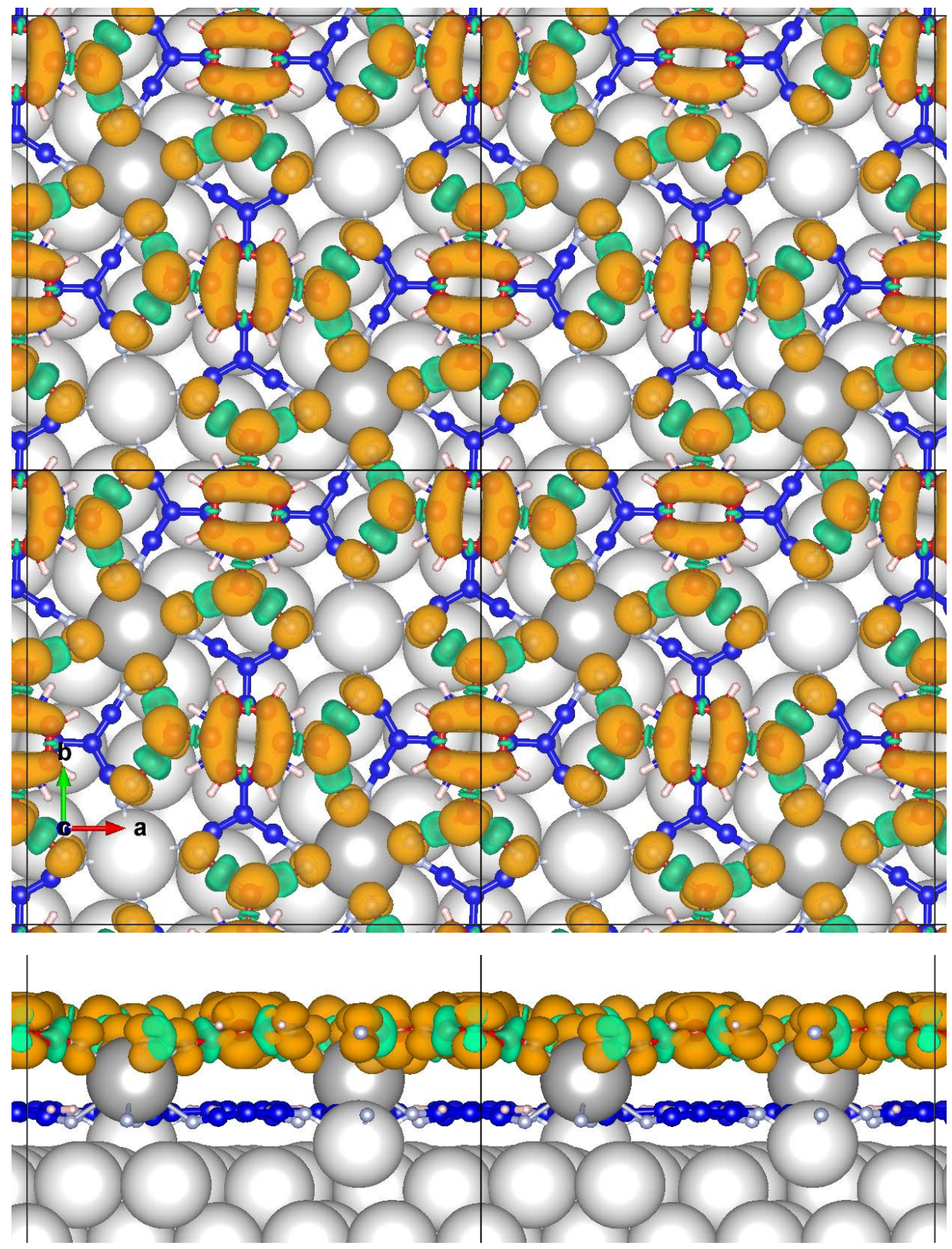

Figure S10: **Spin density of the molecular bilayer including adatoms.** Spin density of the most stable configuration of the molecular bilayer including Pb adatoms using the hybrid HSE06 functional. Orange depicts majority spin density, while green depicts minority spin. The integrated spin moment of the second layer molecules is 0.85 to 0.90 μ_B.

References

1. Frisch, M. J.; Trucks, G. W.; Schlegel, H. B.; Scuseria, G. E.; Robb, M. A.; Cheeseman, J. R.; Scalmani, G.; Barone, V.; Petersson, G. A.; Nakatsuji, H.; Li, X.; Caricato, M.; Marenich, A. V.; Bloino, J.; Janesko, B. G.; Gomperts, R.; Mennucci, B.; Hratchian, H. P.; Ortiz, J. V.; Izmaylov, A. F. *et al.* Gaussian 16 Revision C.01. 2016; Gaussian Inc. Wallingford CT.
